# Presentation

**Published:** 1863-12

**Authors:** 


### PRESENTATION.

An interesting affair took place at the Medical University on Tuesday, in which Professor Sanford Eastman was the recipient of a fine horse, from the class. Death had recently deprived him of that indispensable assistant in professional duties, and afforded the students an opportunity of manifesting their appreciation of, and devotion to, their tutor, than whom the medical profession has no higher ornament, or society a more noble and courteous gentleman. It was a pleasant surprise to the Professor, who was unaware of any such intention on the part of his pupils until the close of his usual anatomical lecture, when it was made known in the following short and impressive speech from Mr. Wm. Pitt Willis, of the class :

Professor Eastman:—In behalf of the students of this University, whom I have the honor to represent, I have been delegated to communicate a small testimony of their regard for you as a faithful friend, a thorough teacher, and above all as a christian physician. We deem it a pleasure to avail ourselves of this rare opportunity of conveying to you in a tangible form the evidence of our profound gratitude. You have labored both in season and out of season to prepare us for the honorable performance of the difficult and responsible duties of the medical profession. Sir, you will please accept this humble token, and with it our unanimous desire that, with health and vigor unimpaired, with your years renewed as the eagle, you may long continue your high prerogative to hold a professional chair in this college, and impart wisdom to each succeeding class whose good fortune it shall be to sit at your feet.

To which the Dean replied:

Gentlemen and Students of the University of Buffalo:—I am entirely taken by surprise, and cannot find words adequate to express my gratitude for this expression of your favor and esteem. You are all aware that for a year past my health has suffered much, and it was with many apprehensions that I should be unable to do justice to you that the present course of lectures was begun. But I thank God that notwithstanding my labors, my health has been, and is still, improving. If my efforts have been crowned with success I can but say, I have done my duty. Let me say for the class, that it has never been my pleasure to meet a class in this college which so far won and merited my esteem for its gentlemanly deportment, intelligence and attention.

At the close, three hearty cheers for Professor Eastman awakened the echoes of the old college halls. The occasion will long be remembered by all who were present. E. H. T.

